# Framework for implementing asylum seekers and refugees’ health into the undergraduate medical curriculum in the United Kingdom

**DOI:** 10.1093/her/cyae002

**Published:** 2024-01-25

**Authors:** Man Jit Jess Kwok, Wright Jacob

**Affiliations:** GKT Medical School, King’s College London, London SE1 1UL, UK; Centre for education, Faculty of Life Science & Medicine, King’s College London, London SE1 1UL, UK

## Abstract

The Ukrainian conflict and the increasing number of asylum seekers and refugees (AS&Rs) in the United Kingdom have highlighted the critical need for a dedicated AS&R health curriculum in medical undergraduate programmes. This study utilized a mixed-method approach to assess the current state of AS&R curricula worldwide, identify shortcomings in the UK’s medical undergraduate curriculum and determine the specific needs of AS&R. A scoping review of literature revealed an absence of comprehensive AS&R health curricula, with many programmes focusing on broader global health issues. Mapping the General Medical Council’s (GMC) Outcomes for Graduates to a UK medical school’s learning outcomes uncovered misalignments with AS&R health requirements and an online survey of AS&R charities exposed barriers related to accessibility and knowledge. The study’s findings emphasize the importance of introducing or reinforcing specific themes in the medical curriculum, such as clarifying terminology and promoting awareness of AS&R organizations. Employing various teaching methods and continuous assessment are vital to evaluate curriculum effectiveness. The development of an AS&R health curriculum is essential to equip future doctors with the necessary skills and knowledge to provide equitable healthcare to this vulnerable population. The study’s findings can serve as a basis for curriculum development and implementation in UK medical schools.

## Introduction

On 24 February 2022, President Putin of the Russian Federation launched a full-scale invasion into the Ukrainian capital, Kyiv. More than 7.8 million refugees fled Ukraine to Europe as of 6 December 2022 [1], with the number expected to rise. The UK’s Home Office estimated a two-fold increase in the proportion of asylum seekers and refugees (AS&Rs) among long-term international migrants, rising from 8% to 16% between 2021 and 2022 [2], owing in part to two new UK Government initiatives to assist Ukrainian refugees.

According to the UN Refugee Convention [3], a refugee is defined as ‘a person who is outside the country of his nationality and is unable or unwilling to avail himself of the protection of that country because of a well-founded fear of being persecuted for reasons of race, religion, nationality, membership in a particular social group, or political opinion’. Trauma-related mental health difficulties are a major health problem associated with AS&R. In addition, many AS&Rs lack access to vaccines, which can significantly increase the number of people who contract infectious diseases. This is due to their congested and unhygienic living conditions, which provide the perfect environment for the development of germs and viruses. As a result of the spontaneity of their departure, AS&Rs frequently have no choice but to remain in inadequate temporary housing, which becomes indefinite because of the Home Office’s backlog of asylum.

The dynamic and growing AS&R population generates new challenges for clinicians; hence, UK’s medical school curriculum ought to ensure that all future doctors are competent to treat such population. Currently, the General Medical Council’s (GMC) Outcomes for Graduates [4], which do not specifically address AS&R, serves as the foundation for UK medical schools’ undergraduate (UG) curricula [4]. As far as we know, substantial literature has been written in the United Kingdom to highlight the needs of an AS&R’s health curriculum, but none has integrated it within UG medical education, even though AS&R’s health is taught as an elective/longitudinally in Germany, United States and Canada. It has been established that a collaborative strategy involving students, teachers and stakeholders from the refugee community will aid in creating an efficient refugee curriculum to deliver equitable healthcare in the United Kingdom [5]. The objective of this paper is to prospectively examine strategies to successfully implement AS&R’s health into UK’s UG medical curriculum. Hence, the corresponding research questions are: (i) What is the suitable framework to design and implement AS&R’s health into the UG medical curriculum in the United Kingdom? (ii) How can AS&R charities contribute development of educational content and knowledge?

## Materials and methods

A comprehensive approach involving multiple phases and mixed methods was employed to gather and assess the viewpoints and insights of organizations dedicated in aiding AS&Rs. The initial phase consisted of a literature scoping review focusing on curricula related to the requirements of AS&Rs in medical education. This was followed by an electronic survey and interviews conducted with key individuals affiliated with charities serving AS&Rs in the United Kingdom. The recruitment of these key individuals was carried out through social media advertisements. The data collection and analysis adhered to General Data Protection Regulation (GDPR) regulations and followed the ethical guidelines established by King’s College London, which granted ethical approval for this research.

### Theoretical frameworks

When exploring appropriate theoretical frameworks for the development of a comprehensive AS&R health curriculum, the biopsychosocial theory [6] stands out as a viable choice. This framework is well-suited to examine what should be taught from the perspectives of AS&R. It allows for the exploration of physical and mental health issues in the biological and psychological domains, while the sociological aspect can delve into social determinants of health affecting physical and mental well-being, as well as the importance of cultural sensitivity. However, it’s important to note that this patient-centred approach does not consider the competencies of clinicians and other healthcare providers.

Another potential framework is Donabedian’s Quality Model [7], which allows for assessment of healthcare quality at three levels: structure (characteristics of healthcare providers, e.g. National Health Service [NHS] administrative processes), process of care (technical competency and interpersonal relationships) and outcomes (AS&R’s healthcare experiences). While this framework aligns with the NHS’s approach, it has a limitation in not establishing causation between process and outcomes. For instance, if the curriculum covers vaccination programmes but doesn’t address the root cause of infectious diseases, such as poor housing, AS&R may still face health risks regardless of vaccinations.

### Phase 1: scoping review

In the process of developing an appropriate curriculum framework, the initial phase involved examining the existing body of literature related to the health of AS&R in contemporary medical education programmes. We opted for a scoping review as our preferred methodology for two primary reasons: firstly, there is a notable lack of comprehensive coverage in this specific field of study, making this method essential for providing an overview of the available literature. Secondly, our study’s goal is not to assess the effectiveness of a particular intervention but rather to explore recurring themes and concepts. Consequently, a systematic review, which is commonly used for evaluating interventions, was not a suitable choice for our purposes.

**Fig. 1.**
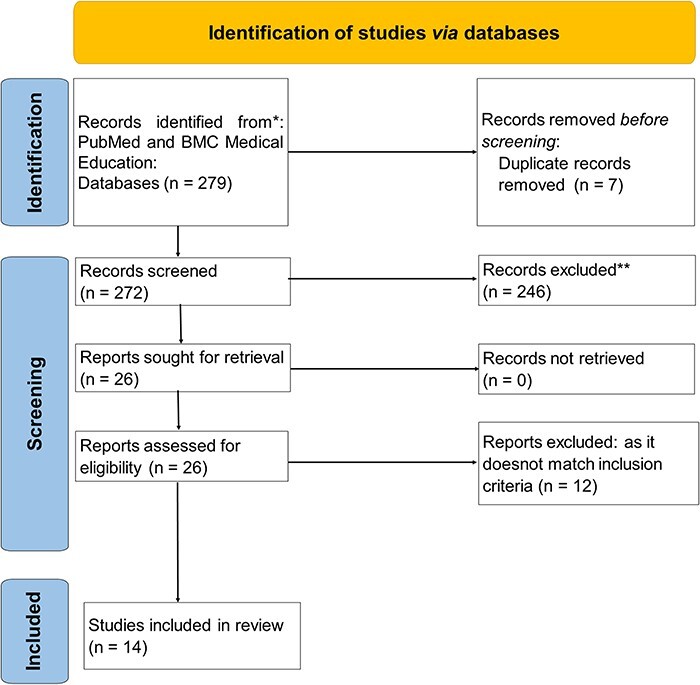
*PRISMA* flow diagram for selection of literature for scoping review.

We followed the Preferred Reporting Items for Systematic Reviews and Meta-Analyses (PRISMA)-ScR [8] review protocol, which acts as a structured checklist for systematically synthesizing evidence. We conducted searches in the PubMed and BMC Medical Education databases for articles published from 2002 to 2022. The flowchart illustrating our process for selecting sources of evidence is available in Fig. 1. We considered studies that employed qualitative, quantitative or mixed-method designs, were written in English and either discussed the creation of a curriculum framework for UG medical education or concentrated on refugee and migrant health education at the UG level.

(see Supplementary Table S1 for search terms used).

Information from the studies was recorded manually, and this includes details such as the title of the study, the author(s), publication year, country of origin and the key findings that pertain to three specific domains: (i) the process of designing a curriculum framework, (ii) the format used for curriculum delivery and (iii) the learning objectives that are directly associated with a curriculum focused on migrant health.

### Phase 2: mapping GMC’s outcomes for graduates against programme learning outcomes of a UK medical school

The scoping review revealed that medical school curricula are typically developed in alignment with established national guidelines, as evidenced by the case of Canada basing its curricula on the seven core competencies defined by the Royal College of Physicians and Surgeons of Canada. There is no common core curriculum for medicine used by all medical schools in the United Kingdom. Instead, the GMC published Outcomes for Graduates [4], which are a set of benchmarks for students to meet by the completion of their UG medical education and this serves as a guide for medical schools as they develop their own curriculum. In this paper, King's College London (KCL)’s UG medical programme will be mapped, since it is one of the universities where graduates will obtain a recognized UK primary medical qualification upon completion of the medical degree.

Second, we compare KCL’s medical programme learning outcomes (PLOs) with the chosen GMC outcomes to assess their alignment. Additionally, we note the year of study when these aligned PLOs are taught. KCL has structured its medical programme into three stages, with stage 1 corresponding to the first year, stage 2 encompassing the second and third years and stage 3 spanning the fourth and fifth years of study. The process of curriculum mapping involves two steps. Firstly, GMC’s outcomes were selected if they are associated with AS&R’s health. The relevance of outcomes was based on the scoping review in Phase 1, where various medical curricula on refugees’ health have been reviewed and specific learning domains have been identified. Secondly, KCL’s medical PLOs were compared against the selected GMC’s outcomes and checked for alignment. Thirdly, the year of study when the aligned PLOs were taught was recorded.

### Phase 3: adjusting learning outcomes to be more specific towards AS&R’s health

While several PLOs have been identified as relevant to an AS&R’s health curriculum, it is important to place a greater emphasis on specific PLOs. This emphasis is necessary either because there is insufficient teaching content associated with them or because they can only be met through specific learning experiences.

Based on the findings of the scoping review, AS&R’s health is often addressed within the broader category of ‘Global Health’. Therefore, in Phase 2, we will cross-reference the GMC’s outcomes with Global Health Learning Outcomes (GHLOs) [9] that directly pertain to AS&R’s health. These GHLOs are recommended by medical organizations approved by the GMC to assist medical schools in developing curricula that focus on specific topics.

### Phase 4: online survey for refugee and migrant charities in the United Kingdom

A crucial initial stage in curriculum design is the identification of needs. In our scoping review, much of the literature we examined were written from the perspective of healthcare providers. To gain insights into the experiences of healthcare from the standpoint of AS&R, we developed an online survey using Google Docs. This survey was distributed to charitable organizations and entities closely involved with AS&R. The aim of this online survey was twofold. Firstly, it sought to capture the experiences of AS&R in healthcare settings. Secondly, it aimed to gather input on the themes and content that should be incorporated into the AS&R curriculum. It is important to note that this new curriculum is intended for use in UK medical schools and should be reflective of the AS&R population specifically within the United Kingdom. (For the complete online survey questionnaire, please refer to Supplementary Table S2).

## Results

### Theoretical framework

The chosen framework is an extension of Donabedian’s Quality Model, known as the Quality Health Outcomes Model [10]. Unlike the linear model of structure-process-outcomes, this dynamic model interconnects four different aspects: system, intervention, client and outcomes. In reference to Donabedian’s model, the system corresponds to the structure, and interventions align with the process. The link between the system and the client (AS&R) implies that AS&R can benefit even without direct intervention, meaning changes in NHS regulations (e.g. simplifying GP registration) can directly impact AS&R. In this context, gaps in the medical curriculum (the ‘system’) will be identified through outcomes mapping, teaching content and delivery methods (the ‘intervention’) will be determined through a scoping review and the healthcare experiences of AS&R (the ‘client’ and ‘outcomes’) will be explored through an online survey. This model aligns well with the curriculum goals because it allows for identifying gaps in the current medical system (through curriculum mapping), directly links interventions to patient populations/issues targeted and keeps the end goal on improving outcomes for AS&R. Additionally, the interconnectedness of the four elements fits with the multi-pronged approach taken to curriculum development. Perspectives from various stakeholders (e.g. charities, AS&R patients) can inform planned content, while evaluation focuses on potential improvements to patient care quality and experiences.

Hence, this dynamic model provides an appropriately comprehensive framework to guide the design and continuous refinement of a curriculum tailor fitted to enhance competencies relevant to caring for the AS&R population.

### Phase 1: scoping review

The scoping review of existing refugee health curricula (Table I) reveals several common themes in the approach to curriculum development, delivery methods and learning objectives. The majority of studies utilized structured frameworks to design their curricula, often beginning with a needs assessment informed by literature reviews and input from stakeholders. Delivery methods emphasized active, experiential learning with direct interactions with refugee patients versus pure didactic teaching. Clinical placements and longitudinal integrations of content were preferred over one-time workshops or electives. Core learning objectives covered cultural awareness, communication abilities, clinical conditions in refugee populations, health systems navigation and social determinants of health. Evaluation methods assessed changes in student knowledge, attitudes and competencies pre- and post-curriculum implementation. In summary, the studies pointed to establishing competency frameworks, prioritizing clinical experiences and measuring acquisition of skills essential for caring for refugee patients. The common aim was graduating clinicians equipped to deliver culturally sensitive, patient-centred care to this vulnerable population.

**Table I.** Results of scoping review

Title		Country of research	Key findings
Refugee health and medical student training	[11]	United States	Design process Primary steps include literature review and assessment on the curriculum providers’ experiences. Expectations from different stakeholders were also explored through small focus groups. Clear goals were further defined. Delivery methods Monthly sessions where students take histories from refugees and screen for mental health illnesses.
Effects of a refugee elective on medical student perceptions	[12]	United States	Learning objectives UN guidelines and understanding how one’s personal factors can impact their ability to practice medicine.
Developing a curriculum framework for global health in family medicine: emerging principles, competencies and educational approaches	[13]	Canada	Design process Four steps: (i) setting a mission statement, (ii) defining the scope of the topic, (iii) describing core principles and (iv) exploring different learning approaches.
Introducing global health into the undergraduate medical school curriculum using an e-learning programme: a mixed method pilot study	[14]	Canada	Design process Randomized controlled trial method was used to measure difference of knowledge between those using e-learning (test group) and PDF articles (control group).
A collaborative clinical and population-based curriculum for medical students to address primary care needs of the homeless in New York City shelters	[15]	United States	Design process To evaluate the impact of curriculum, a paired *t*-test was performed to analyse students’ knowledge, attitude and self-efficacy towards the topic pre- and post-curriculum. Delivery method Clinical-based teachings conducted in local communities.
UK medical education on human trafficking: assessing uptake of the opportunity to shape awareness, safeguarding and referral in the curriculum	[16]	United Kingdom	Learning objectives Use specific terminology, such as ‘human trafficking’, instead of simply including it under umbrella terms, such as groups that need ‘safeguarding’. Delivery method Self-directed e-learning and shadowing health care workers.
The refugee health partnership: a longitudinal experiential medical student curriculum in refugee/asylee health	[17]	United States	Design process Evaluation of the programme is done via two surveys, a quantitative self-reported one and a qualitative one. Learning objectives Four main objectives were identified: (i) effective communication, (ii) patient-centred care in practice, (iii) use narrative medicine as a healing method and (iv) promote best practices through designing a guide for healthcare professions when interacting with refugees and by organizing teachings on barriers of accessing healthcare. Delivery method The core of the programme is to form a mutually beneficial relationship between resettled refugees and medical students; hence, the curriculum is skills- and service-based. On top of home visits, students attend compulsory teaching sessions.
A student-led curriculum framework for homeless and vulnerably housed populations	[18]	Canada	Delivery method The majority of students expressed significantly higher interest in community outreach and direct interactions with patients as opposed to lectures.
Global health education in medical schools (GHEMS): a national, collaborative study of medical curricula	[19]	United Kingdom	Learning objectives Broader understanding of private and international healthcare organizations apart from NHS. In addition, there should be focus on how climate change can impact on global health security.
Caring for refugees and asylum seekers in Canada: early experiences and comprehensive global health training for medical students	[20]	Canada	Learning objectives Demonstrating cross-cultural communications and providing both culture- and age-appropriate care. Hence, a module focusing on elderly refugee health, which includes culturally sensitive palliative care, polypharmacy, etc. As well as modules on understanding other marginalized groups, such as LGBTQ + . Delivery method Theoretical learning via e-learning, followed by community service-based practical learning.
Refugee health curriculum in undergraduate medical education (UME): a scoping review	[21]	Canada	Learning objectives Cross-cultural awareness and overcoming language barriers are the main recurring themes. Other learning outcomes include advocacy skills, particularly preparing affidavits and status applications. Delivery methods Fewer didactic sessions and more active learning methods, such as clinical placements, case-based discussions and simulation exercises. One key finding is that refugee health is often an elective or a one-off workshop, which is not effective in allowing students to retain knowledge. Other delivery methods suggestions include exchange programmes, collaborating with refugee advocacy or resettlement agency.
The design and implementation of a longitudinal social medicine curriculum at the University of Vermont’s Larner College of Medicine	[22]	United States	Design process Uses Kern’s six-step model for curriculum development, Delivery method Students were responsible to announce the new theme every week to encourage participation.
Health and medical care for refugees: design and evaluation of a multidisciplinary clinical elective for medical student	[23]	Germany	Design process Needs were identified from the perspective of health care providers through self-reporting on practical skills essential for clinic. There were three major components: theoretical, practical and reflective. Delivery method Theory taught through lectures by range of healthcare workers; practical are placement opportunities in local outpatient clinics; reflection done through case-based discussions.
An undergraduate medical education framework for refugee and migrant health: curriculum development and conceptual approaches	[24]	Canada	Design process Curriculum framework was based on the seven essential competencies outlined by the Royal College of Physicians and Surgeons of Canada. Interviews on Canadian medical schools were conducted to identify any existing refugee health-related framework; follow-up surveys are then sent out based on the interview contents. Delivery method Clinical experiences include workshops with standardized patients, and placements working with settlement agencies.

### Phase 2: mapping GMC’s outcomes for graduates against PLOs of a UK medical school

Out of the 153 outcomes outlined by the GMC, 88 have been identified as relevant to the health of AS&R. KCL has structured its medical PLOs in a way that incorporates all the GMC’s outcomes directly into their curriculum without any alterations. In addition to these 88 outcomes, KCL has introduced an additional set of 32 PLOs. Most of these PLOs are integrated throughout the entire medical curriculum, spanning both Stages 2 and 3. However, there are five PLOs that are exclusively taught during Stage 3, and one PLO that is specifically covered during Stage 2. (See Supplementary Table S3 for full curriculum mapping).

### Phase 3: Adjusting learning outcomes to be more specific towards AS&R’s health

After a thorough analysis in Phase 2, it was found that 27 out of the 88 outcomes defined by the GMC were closely associated with the health of AS&R when compared with the GHLO. It is important to note that with the statement ‘demonstrating awareness of the complexity of global health governance, including the roles of international organisations, the commercial sector, and civil society’ does not align with any of the GMC’s outcomes. This discrepancy arises because the GHLOs are derived from Tomorrow’s Doctors, which is the previous edition of Outcomes for Graduates. Furthermore, several GHLOs are connected to fewer than three GMC outcomes. (For the detailed mapping of GMC outcomes to GHLO, please refer to Supplementary Table S4).

### Phase 4: Online survey for refugee and migrant charities in the United Kingdom

All five responses we received came from either local or international charities with presence in the United Kingdom. According to the survey results, the health conditions encountered by AS&R can be categorized into two groups based on the responses: (i) pre-existing conditions, including issues like post-traumatic stress disorder; (ii) conditions that develop after their arrival in the United Kingdom, such as skin lesions resulting from inadequate living conditions.

Regarding the obstacles in healthcare access and potential solutions, all respondents highlighted the insufficient understanding of the UK healthcare system, particularly regarding refugees’ entitlement, both among healthcare users and providers. Language barriers were also consistently mentioned, with three responses focusing on this issue. The proposed solutions to address these barriers, as suggested by clinicians, include: (i) self-education among healthcare providers about NHS charging criteria, social determinants of health and cultural awareness; (ii) streamlining the healthcare access process to make it more accessible; (iii) emphasizing the importance of defining terms and recognizing the diversity within the AS&R population.

**Fig. 2.**
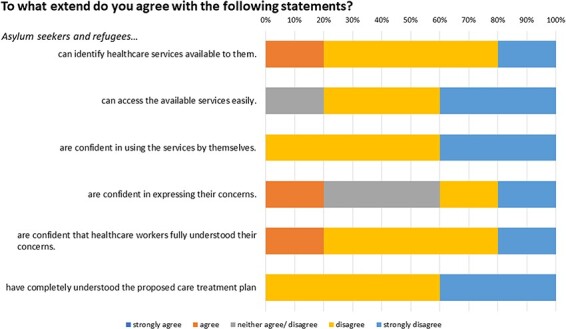
Data compiled from an online survey distributed to charitable organizations serving refugee populations. Diagram represents insights from Question 5 of the survey.

**Fig. 3.**
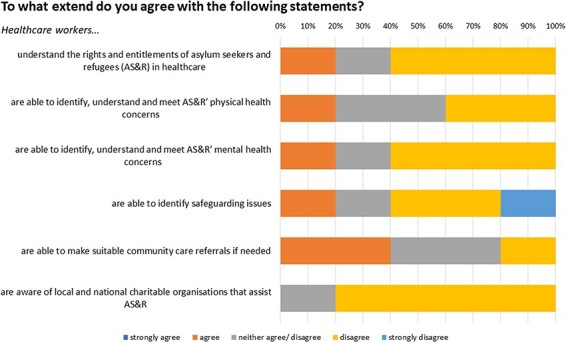
Data compiled from an online questionnaire distributed to charitable organizations serving refugee populations. Diagram represents insights from Question 6 of the survey.

Figures 2 and 3 highlight the refugees’ confidence in healthcare and healthcare workers’ competence. The results were measured on a 5-point Likert scale, indicating that most respondents disagreed with statements about AS&Rs’ proficiency in using healthcare services. Moreover, the responses also indicated disagreement with statements concerning healthcare workers’ adequate knowledge.

## Discussion

As the number of AS&R in the United Kingdom continues to rise, it becomes increasingly important to educate future doctors on how to deliver effective healthcare to these vulnerable populations. While the original focus was on immigrants, it’s worth noting that most of the literature pertaining to health education in medical curricula for migrants primarily centres on AS&R. One possible explanation for this emphasis is that AS&Rs are often seen as particularly vulnerable, given that their decision to leave their home country is typically involuntary and unplanned. Consequently, the focus of this paper has shifted to address the unique healthcare needs and challenges faced by AS&R.

To develop a curriculum framework, we employed Quality Health Outcomes Model, which is specifically tailored for medical curricula and has been consistently utilized in multiple studies within our scoping review. When considering the integration of a new curriculum into the existing content-heavy UG medical curriculum, it’s essential to establish its significance. Insights from the online survey revealed that AS&Rs generally have negative experiences with the NHS, primarily due to two core issues: (i) inaccessibility: this is primarily rooted in communication barriers, which encompass not only language differences but also cultural disparities; (ii) lack of understanding: AS&Rs often struggle to grasp their healthcare rights and the relevant regulations. Refugee organizations stressed the need for doctors to exhibit greater sensitivity to multiculturalism and make increased use of translation services. Interestingly, despite open communication and recognition of cultural determinants of health being consistently emphasized throughout the entire medical curriculum and mentioned in various sections of the GMC’s ‘Outcomes for Graduates’, there is still a perceived gap in this area. Another noteworthy point raised was the potential safeguarding issues associated with using family members as interpreters. While this concern was mentioned both in the GHLO and in responses from refugee charities, it is not addressed in any of the studies reviewed. The GMC’s outcomes [4] merely state the need to ‘identify signs and symptoms of abuse or neglect, and be able to safeguard children, young people, adults, and older people’, which could be challenging if the individual mistreating the patient is also acting as the translator.

The scoping review analysed numerous studies from the United States, Canada and Germany, which also highlighted the issue of inaccessibility in healthcare for AS&Rs. However, the online survey specifically identified a problem unique to the United Kingdom, which is healthcare workers’ lack of knowledge about refugees’ entitlements. This issue encompasses two main challenges: (i) the complex GP registration system, which affects the accessibility of primary care services, as GPs are usually the first point of contact for patients, and GP referrals are often necessary for accessing secondary care provided by hospital specialists; (ii) overcharging within the NHS, which can deter AS&Rs from seeking healthcare. The GMC outcomes [4] are concise and touch upon principles of equality law in patient care but do not specifically address the right to healthor the understanding of treatment costs. Following the needs assessment, the next step is to define the learning objectives of the curriculum, as presented in Table II. Drawing from the GHLO, certain themes were identified as essential but currently not covered in the GMC’s outcomes. Additionally, some themes require greater emphasis and specificity within the existing UG medical curriculum. Certain GMC outcomes related to AS&R are vague and may be overlooked in the core curriculum. Therefore, the subsequent step in Quality Health Outcomes Model involves selecting delivery methods that ensure alignment between the planned curriculum and the content students have already encountered. While theoretical-based teaching approaches are more common, clinical-based learning is more effective in establishing a mutually beneficial relationship between AS&R, who require high-quality healthcare, and medical students, who gain knowledge by providing healthcare. All participants in the AS&R health curriculum, including refugee charity and resettlement organizations, medical schools, students and patients, should contribute to educational methods. The final step in curriculum development is evaluating the framework. This can be done statistically through a paired *t*-test of students’ knowledge and attitudes before and after completing the course. Additionally, ongoing reflection and self-assessment by students throughout the curriculum can be valuable.

**Table II.** Themes to include in the AS&R’s health curriculum

Themes to be newly introduced	Themes to be reinforced
Universal and UK’s definitions of AS&R AS&R’s population in the United Kingdom Population demographics, including nationality, what they are persecuted for, resettlement areas in the United Kingdom. Cultural awareness of identified nationalities, such as languages, religion, medical practices, vaccinations National and international organizations and charities that assist AS&R in clinical and non-clinical ways, e.g. housing, employment, education. Advocating for AS&R Supporting documentation that can help resettlement	Utilizing translational services and associated potential safeguarding issues. Patients’ confidentiality, particularly scenarios where clinicians are legal obligation to report to Home Office GP registration process Healthcare rights of AS&R in primary, secondary and tertiary care NHS’s regulations on overseas visitor treatment charging and exempted populations.

There are several limitations to this study. Firstly, the themes presented in Table II are not exhaustive. They were identified after mapping KCL’s UG medical curriculum to the GMC’s outcomes and further extrapolating from GHLO. Ideally, the curricula of all 35 medical schools should be mapped, and learning outcomes from various medical branches, such as public health and emergency medicine, should be considered. Another shortcoming is the limited number of responses from refugee charities, which prevented statistical analysis of the online survey due to uncertainties and low significance levels.

Future developments in this study should include designing all the learning outcomes for an AS&R curriculum and trialling its implementation in one of the UK’s medical schools. As the impact of a new curriculum may not be immediately observed, resources can be created virtually or physically for clinicians to refer to when dealing with patients from these populations.

## Conclusion

In conclusion, this paper demonstrates the need for and provides a framework to integrate education on AS&R health into UG medical curricula in the United Kingdom. Through a multi-phase approach involving literature review, curriculum mapping, survey of refugee organizations and application of an appropriate theoretical model, key gaps were identified in existing medical training related to care for this vulnerable population. The proposed curriculum aims to enhance competencies in areas like cultural awareness, health systems navigation and social determinants of health. Successful implementation requires adaptation to national guidelines, experiential learning methods, stakeholder collaboration and continuous evaluation. If enacted properly, this has potential to significantly improve new physicians’ preparation to deliver equitable, patient-centred care to AS&Rs. Limitations of this study include incomplete curriculum mapping across all UK medical schools and insufficient survey responses. Next steps would involve finalizing learning outcomes, piloting the curriculum’s delivery and measuring its impacts on learner knowledge, attitudes and clinical skills. Overall, this project provides a strong foundation and model to guide integration of AS&R health education into UG medical programmes. Doing so will help fulfil the profession’s commitment to health equity and ensure all patients in the United Kingdom, regardless of background, receive high-quality care suited to their unique needs.

## Supplementary Material

cyae002_Supp
