# Supplementary material for: Framework for implementing asylum seekers and refugees’ health into the undergraduate medical curriculum in the United Kingdom
Source: Health Educ Res. 2024 Jan 25;39(2):170–81. doi: 10.1093/her/cyae002 (PMC10952400; doi:10.1093/her/cyae002)
Supplement: cyae002_Supp [file cyae002_supp.zip › Supp/Spplementary 2 .docx]

Supplementary table 2 : Online Survey Questionnaire.

Understanding Charities' Perspective on Refugees and Asylum Seekers' Healthcare Experiences

Thank you for taking part in my research project focused on forming a framework for implementing refugee and asylum seekers' health into the undergraduate medical curriculum in the United Kingdom.

The purpose of the survey is to understand the gaps in healthcare for refugees and asylum seekers through the perspectives of charities that work directly with their communities.

This will take you approximately 10 minutes to complete.

* Indicates required question

1. Email *
2. What are the most common physical and mental health conditions refugees and asylum seekers in the UK present with? *
3. From your observations, what are the main problems with physicians that inhibit refugees and asylum seekers from receiving suitable healthcare in primary care *

settings?

*(e.g. language barrier, cultural incompetence, lack of knowledge on refugee rights, lack of confidence in approaching sensitive topics, lack of knowledge on impacts of social determinants on health, etc.)*

1. What essential points physicians must know and be aware of when consulting and treating refugees and asylum seekers patients? *
2. What should be included in the medical curriculum (i.e. university course for medical students) regarding refugees' and asylum seekers’ health? *

From your observations, to what extent do you agree with the following statements?

# Refugees and asylum seekers… *

*Mark only one oval per row.*

strongly agree agree neither agree/ disagree disagree strongly disagree

## can identify healthcare services available to them.

**can access the available services easily.
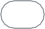
**
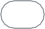

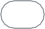

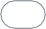

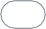
 **are confident in using the services by**

##
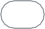

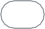

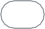

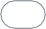

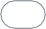

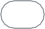

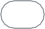

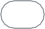

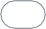

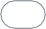
themselves.


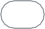

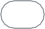

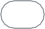

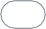

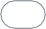
**are confident in expressing their concerns.
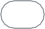
**
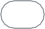

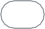

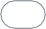

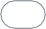
 **are confident that healthcare workers fully**

##
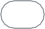

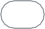

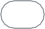

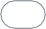

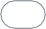
understood their concerns.

**have completely understood the proposed care treatment plan (including the reason for examinations, tests, medications, vaccinations etc.)**

#
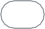

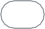

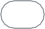

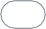

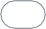

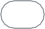

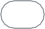

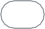

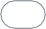

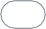
Healthcare workers (e.g. doctors, nurses, healthcare assistants) … *

| *Mark only one oval per row.* | strongly agree | agree | neither agree/ disagree | disagree | strongly disagree |
| --- | --- | --- | --- | --- | --- |
| **understand the rights and entitlements of** |  |  |  |  |  |
| **refugees and asylum seekers in healthcare (e.g.** |  |  |  |  |  |
| **temporary registration in GP, free of charge to** |  |  |  |  |  |
| **primary care services, etc)** |  |  |  |  |  |
| **are able to identify, understand and meet** |  |  |  |  |  |
| **refugees and asylum seekers’ physical health** | 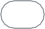 | 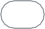 | 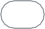 | 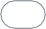 | 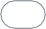 |
| **concerns.** |  |  |  |  |  |
| **are able to identify, understand and meet** |  |  |  |  |  |
| **refugees and asylum seekers’ mental health** | 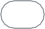 | 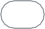 | 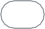 | 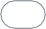 | 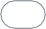 |
| **concerns.** |  |  |  |  |  |
| **are able to identify safeguarding issues (sexual** |  |  |  |  |  |
| **exploitation, human trafficking, financial** |  |  |  |  |  |
| **abuse, domestic abuse, female genital** |  |  |  |  |  |
| **mutilation, etc.)** |  |  |  |  |  |
| **are able to make suitable community care** |  |  |  |  |  |
| **referrals if needed.** |  |  |  |  |  |
| **are aware of local and national charitable** |  |  |  |  |  |
| **organisations that assist refugees and asylum** | 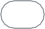 | 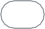 | 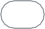 | 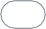 | 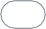 |
| **seekers.** |  |  |  |  |  |

**Thank you for completing this survey.**

If you have any questions or would like to read the end result of my research project, please feel free to contact me via [jess.kmj@gmail.com.](mailto:jess.kmj@gmail.com)


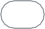

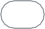

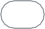

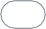

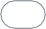
This content is neither created nor endorsed by Google.

Forms
