# Supplementary material for: Framework for implementing asylum seekers and refugees’ health into the undergraduate medical curriculum in the United Kingdom
Source: Health Educ Res. 2024 Jan 25;39(2):170–81. doi: 10.1093/her/cyae002 (PMC10952400; doi:10.1093/her/cyae002)
Supplement: cyae002_Supp [file cyae002_supp.zip › Supp/Supplementary 3.docx]

# **Supplementary table 3. Mapping KCL GKT Medical School Programme Learning to GMC Learning Outcomes**

*PLOs that do not have corresponding GMC outcomes are highlighted in blue*

| **Number of Corresponding GMC’s Outcomes** | **Programme Learning Outcomes of KCL’s medical curriculum** | **Stages where the PLOs are taught** |
| --- | --- | --- |
| 1 | 1) demonstrate appropriate generic personal and professional values and behaviours. Keep to the GMC’s good medical practice and the explanatory guidance | 1, 2, 3 |
| 2a | 2) demonstrate the clinical responsibilities and role of the doctor | 1, 2 |
|  | 4) demonstrate understanding and behaviour in accordance with the principles of the NHS Constitution both at work and, as a respected professional, in all aspects of life | 1, 2, 3 |
| 2b | 5) demonstrate compassionate professional behaviour and their professional responsibilities in making sure the fundamental needs of patients are addressed | 2, 3 |
| 2c | 6) summarise the current ethical dilemmas in medical science and healthcare practice; the ethical issues that can arise in everyday clinical decision-making; and apply ethical reasoning to situations which may be encountered in the first years after graduation | 1, 2, 3 |
|  | 8) identify groups that experience more health inequality (such as the homeless, prison, looked after children, severe mental illness, refugees, asylum seekers and undocumented migrants) and be aware of the common health problems that these groups experience | 1, 2, 3 |
| 2d | 9) maintain confidentiality and respect patients’ dignity and privacy | 2, 3 |
|  | 10) outline the law relating to the capacity of children and young people to consent to and refuse treatment, and the ethical implications. Explain the respective roles of parents/ guardians, healthcare professionals and the courts in best interests decisions about the treatment of children and young people | 1, 2, 3 |
|  | 11) recognises and works within the principles of patient confidentiality, recognising when it is justifiable or mandatory to disclose confidential information and how to do this appropriately. Demonstrate awareness of good practice in sharing information appropriately with relatives, carers and other relevant advocates | 1, 2, 3 |
| 2e | 12) act with integrity, be polite, considerate, trustworthy & honest | 1, 2, 3 |
| 2f | 13) take personal and professional responsibility for their actions | 1, 2, 3 |
| 2h | 15) recognise and acknowledge their own personal and professional limits and seek help from colleagues and supervisors when necessary, including when they feel that patient safety may be compromised | 1, 2, 3 |
| 2i | 16) protect patients from any risk posed by their own health including: * the risks to their health and to patient safety posed by self-prescribing medication and substance misuse  *the risks to their health and to patient safety posed by fatigue – they must apply strategies to limit the impact of fatigue on their health. | 2, 3 |
| 2j | 17) recognise the potential impact of their attitudes, values, beliefs, perceptions and personal biases (which may be unconscious) on individuals and groups and identify personal strategies to address this | 2, 3 |
|  | 18) works with patients and carers according to the ethical, legal and professional responsibilities of the doctor, in the context of a diverse and multicultural society. Demonstrates an awareness of protected characteristic and potential barriers for individual patients and carers | 1, 2, 3 |
|  | 19) be able to recognise, priorities and reflect on what the patient and their loved ones need as human beings | 2, 3 |
| 2k | 20) demonstrate the principles of person-centred care and include patients and, where appropriate, their relatives, carers or other advocates in decisions about their healthcare needs | 2, 3 |
| 2l | 21) explain and demonstrate the importance of:  *seeking patient consent, or the consent of the person who has parental responsibility in the case of children and young people, or seeking the views of those with lasting power of attorney or independent mental capacity advocates if appropriate  *providing information about options for investigations, treatment and care in a way that enables patients to make decisions about their own care  *assessing the mental capacity of a patient to make a particular decision, including when the lack of capacity is temporary, and knowing when and how to take action | 2, 3 |
|  | 22) demonstrate an understanding of the concept of informed consent/ able to explain consent process for common interventions and recognises when obtaining consent is particularly challenging (eg contraception in children less than 18) | 1, 2, 3 |
| 2m | 23) act appropriately, with an inclusive approach, towards patients and colleagues | 2, 3 |
| 2o | 25) raise and escalate concerns through informal communication with colleagues and through formal clinical governance and monitoring systems about:  *patient safety and quality of care  *bullying, harassment and undermining | 2, 3 |
| 4 | 38) demonstrate knowledge of the principles of the legal framework in which medicine is practised in the jurisdiction in which they are practising, and have awareness of where further information on relevant legislation can be found. | 2, 3 |
|  | 42) able to recognise, analyse and act using knowledge and understanding of ethical and legal principles governing medical practice, and be able to justify those actions | 1, 2, 3 |
| 5a | 46) place patients’ needs and safety at the centre of the care process | 2, 3 |
| 5b | 47) promote and maintain health and safety in all care settings and escalate concerns to colleagues where appropriate, including when providing treatment and advice remotely | 2, 3 |
| 5d | 52) apply measure to prevent the spread of infection, and apply the principles of infection prevention & control | 1, 2, 3 |
| 6a | 58) recognise the complex medical needs, goals and priorities of patients, the factors that can affect a patient’s health and wellbeing and how these interact. These include psychological and sociological considerations that can also affect patients’ health | 2, 3 |
|  | 59) understand that mind and body interact, and the needs of people experiencing long term conditions and mental health problems. Be able to recognise, and respond appropriately, to a mental health problem in someone in hospital with a ‘body problem’ and to a physical condition in someone in hospital/ clinic with a mental health problem | 2, 3 |
|  | 60) recognises how a patient’s pre-existing medical/ psychological conditions may impact on their presentation for a different condition | 2, 3 |
| 6b | 61) identify the need to adapt management proposals and strategies for dealing with health problems to take into consideration patients’ preferences, social needs, multiple morbidities, frailty and long term physical and mental conditions | 2, 3 |
| 6c | 62) demonstrate working collaboratively with patients, their relatives, carers or other advocates, in planning their care, negotiating and sharing information appropriately and supporting patient self-care | 2, 3 |
|  | 63) demonstrate the ability to work with the patient to help them see their strength and vulnerabilities to improve their capacity to improve or manage their health | 2, 3 |
| 6d | 64) demonstrate working collaboratively with other health and care professionals and organisations when working with patients, particularly those with multiple morbidities, frailty and long term physical and mental conditions | 2, 3 |
| 6e | 65) recognise how treatment and care can place an additional burden on patients and make decisions to reduce this burden where appropriate, particularly where patients have multiple conditions or are approaching the end of life | 2, 3 |
|  | 66) can prioritise decision-making that improves patient health and wellbeing | 2, 3 |
| 7a | 70) identify signs and symptoms of abuse or neglect and be able to safeguard children, young people, adults and older people, using appropriate systems for sharing information, recording and raising concerns, obtaining advice, making referrals and taking action71) take a history that includes consideration of the patient’s autonomy, views and any associated vulnerability, and reflect this in the care plan and referrals | 2, 3 |
| 7b | 71) take a history that includes consideration of the patient’s autonomy, views and any associated vulnerability, and reflect this in the care plan and referrals | 2, 3 |
| 7c | 72) assess the needs of and support required for children, young people and adults and older people who are the victims of domestic, sexual or other abuse | 2, 3 |
| 7e | 74) assess the needs of, and support required, for people with mental health conditions | 2, 3 |
| 7f | 75) adhere to the professional responsibilities in relation to procedures performed for non-medical reasons, such as female genital mutilation and cosmetic interventions | 3 |
| 7g | 76) explain the application of health legislation that may result in the deprivation of liberty to protect the safety of individuals and society | 2, 3 |
| 7h | 77) recognise where addiction (to drugs, alcohol, smoking or other substances), poor nutrition, self- neglect, environmental exposure, or financial or social deprivation are contributing to ill health. And take action by seeking advice from colleagues and making appropriate referrals | 2, 3 |
| 7i | 78) describe the principles of equality legislation in the context of patient care. | 2, 3 |
| 9a | 83) demonstrate their contribution to effective interdisciplinary team working with doctors from all care settings and specialties, and with other health and social care professionals for the provision of safe and high-quality care | 2, 3 |
|  | 84) can define clinical governance in F1 clinical context. Describe the role of the Caldicott Guardian and recognises when to seek their involvement | 2, 3 |
| 9b | 85) work effectively with colleagues in ways that best serve the interests of patients. This includes: safely passing on information using clear and appropriate spoken, written and electronic communication | 2, 3 |
| 9c | 86) recognise and show respect for the roles and expertise of other health and social care professionals and doctors from all specialties and care settings in the context of working and learning as a multi- professional team. | 2, 3 |
| 10a | 87) communicate clearly, sensitively and effectively with patients, their relatives, carers or other advocates, and colleagues from medical and other professions, by:  *listening, sharing and responding  *demonstrating empathy and compassion demonstrating effective verbal and non-verbal interpersonal skills  *making adjustments to their communication approach if needed, for example for people who communicate differently due to a disability or who speak a different first language  *seeking support from colleagues for assistance with communication if needed | 2, 3 |
|  | 88) demonstrates a patient-centred approach to care. Communicates clearly, sensitively and honestly with patients, families and carers. Recognises that the patient’s perspective and dignity is critical to all clinical encounters. Able to adapt the language of the consultation to the audience and situation | 1, 2, 3 |
| 10b | 89) communicate by spoken, written and electronic methods (including in medical records) clearly, sensitively and effectively with patients, their relatives, carers or other advocates, and colleagues from medical and other professions. This includes, but is not limited to, the following situations: *where there is conflict or disagreement  *when communicating with children and young people  *when English is not the patient’s first language - by using an interpreter, translation service or other online methods of translation  *when advocating for patients’ needs *when making referrals to colleagues from medical and other professions *when providing care remotely, such as carrying | 2, 3 |
| 10c | 90) use methods of communication used by patients and colleagues such as technology-enabled communication platforms, respecting confidentiality and maintaining professional standards of behaviour | 2, 3 |
| 11a | 93) elicit and accurately record a patient’s medical history, including family and social history, working with parents and carers or other advocates when the patient is a child or young person or an adult who requires the support of a carer or other advocate | 2, 3 |
|  | 94) Undertakes exploration of a patient’s social support networks to help recovery/ health management, and provides information around local or national support groups that may be useful, or signposts others who may fulfil this role (eg health navigators) | 2, 3 |
| 11b | 95) encourage patients’ questions, discuss their understanding of their condition and treatment options, and take into account their ideas concerns, expectations, values and preferences | 2, 3 |
|  | 96) demonstrate ability to assess a patient’s desire and readiness to change | 2, 3 |
| 11c | 97) acknowledge and discuss information patients have gathered about their conditions and symptoms, taking a collaborative approach | 2, 3 |
| 11d | 98) provide explanation, advice and support that matches patients’ level of understanding and needs, making reasonable adjustments to facilitate patients’ understanding if necessary | 2, 3 |
| 11f | 102) work with patients, or their legal advocates, to agree how they want to be involved in decision making about their care and treatment | 2, 3 |
| 12 | 104) newly qualified doctors must work collaboratively with patients and colleagues to diagnose and manage clinical presentations safely in community, primary and secondary care settings and in patients’ homes. Newly qualified doctors must, wherever possible, support and facilitate patients to make decisions about their care and management. | 2, 3 |
|  | 105) implements the red flag system when taking a history to ensure issues of patient safety are ‘caught’ | 2, 3 |
|  | 107) recognise how common Medically Unexplained Physical Symptoms are, presenting in General Practice and many hospital outpatient clinics and how to support and believe patients’ physical symptoms whilst helping them to understand there is no physical cause | 2, 3 |
|  | 109) demonstrates understanding of the role of continuity of care and enablers and barriers to this | 2, 3 |
|  | 145) demonstrate basic knowledge, skills and attitudes of cultural competence, including the need for patient dignity and perspective and the impact of cultural practices on health | 2, 3 |
| 14a | 146) propose an assessment of a patient’s clinical presentation, integrating biological, psychological and social factors, agree this with colleagues and use it to direct and prioritise investigations and care | 2, 3 |
| 14b | 147) safely and sensitively undertake: *an appropriate physical examination (with a chaperone present if appropriate)  *a mental and cognitive state examination, including establishing if the patient is a risk to themselves or others, seeking support and making referrals if necessary  *a developmental examination for children and young people | 2, 3 |
| 14c | 157) interpret findings from history, physical and mental state examinations | 2, 3 |
| 14d | 158) propose a holistic clinical summary, including a prioritised differential diagnosis/diagnoses and problem list | 2, 3 |
| 14e | 159) propose options for investigation, taking into account potential risks, benefits, cost effectiveness and possible side effects and agree in collaboration with colleagues if necessary, which investigations to select | 2, 3 |
| 14f | 160) interpret the results of investigations and diagnostic procedures, in collaboration with colleagues if necessary | 2, 3 |
| 14g | 161) synthesise findings from the history, physical and mental state examinations and investigations, in collaboration with colleagues if necessary, and make proposals about underlying causes or pathology | 2, 3 |
| 14h | 162) understand the processes by which doctors make and test a differential diagnosis and be prepared to explain their clinical reasoning to others | 2, 3 |
| 14i | 163) make clinical judgements and decisions with a patient, based on the available evidence, in collaboration with colleagues and as appropriate for their level of training and experience, and understand that this may include situations of uncertainty | 2, 3 |
|  | 164) recognise the limitations of clinical studies and become able to explain those to patients in order to reach agreement with regard to the investigations, management and prevention of common diseases | 2, 3 |
| 14j | 165) take account of patients’ concerns, beliefs, choices and preferences, and respect the rights of patients to reach decisions with their doctor about their treatment and care and to refuse or limit treatment | 2, 3 |
| 14k | 166) seek informed consent for any recommended or preferred options for treatment and care | 2, 3 |
| 14l | 167) propose a plan of management including prevention, treatment, management and discharge or continuing community care, according to established principles and best evidence, in collaboration with other health professionals if necessary | 2, 3 |
| 14m | 168) support and motivate the patient’s self-care by helping them to recognise the benefits of a healthy lifestyle and motivating behaviour change to improve health and include prevention in the patient’s management plan | 2, 3 |
|  | 170) recognise the differences between mental illness and the range of normal responses to stress and life events (including bereavement and grief), and how this may manifest in different personalities | 2, 3 |
|  | 171) can prioritise decision-making that improves patient health and wellbeing | 2, 3 |
| 15 | 172) demonstrate that they can make appropriate clinical judgements when considering or providing compassionate interventions or support for patients who are nearing or at the end of life. They must understand the need to involve patients, their relatives, carers or other advocates in management decisions, making referrals and seeking advice from colleagues as appropriate. | 2, 3 |
| 18a | 180) establish an accurate medication history, covering both prescribed medication and other drugs or supplements, and establish medication allergies and the types of medication interactions that patients experience | 1, 2, 3 |
| 18b | 181) carry out an assessment of benefit and risk for the patient of starting a new medication taking into account the medication history and potential medication interactions in collaboration with the patient and, if appropriate, their relatives, carers or other advocates | 2, 3 |
| 18c | 183) provide patients, their relatives, carers or other advocates, with appropriate information about their medications in a way that enables patients to make decisions about the medications they take | 2, 3 |
| 18d | 184) agree a medication plan with the patient that they are willing and able to | 2, 3 |
| 18e | 185) access reliable information about medications and be able to use the different technologies used to support prescribing follow | 1, 2, 3 |
| 18h | 189) describe the role of clinical pharmacologists and pharmacists in making decisions about medications and prescribe in consultation with these and other colleagues as appropriate | 2, 3 |
| 18i | 190) communicate appropriate information to patients about what their medication is for, when and for how long to take it, what benefits to expect, any important adverse effects that may occur and what follow-up will be required | 2, 3 |
| 18m | 194) respect patient choices about the use of complementary therapies, and have a working knowledge of the existence and range of these therapies, why patients use them, and how this might affect the safety of other types of treatment that patients receive | 3 |
| 18n | 195) recognise the challenges of delivering these standards of care when prescribing and providing treatment and advice remotely, for example via online services | 3 |
| 18o | 196) recognise the risks of over-prescribing and excessive use of medications and apply these principles to prescribing practice | 2, 3 |
|  | 198) recognises and can discuss with a patient the need/ desire to stop medication and the likely consequences, and the process of deprescribing | 2, 3 |
| 19b | 209) apply the requirements of confidentiality and data protection legislation and comply with local information governance and storage procedures when recording and coding patient information | 2, 3 |
|  | 210) understand legal requirements and demonstrate appropriate behaviours in storing and retrieving information, working within the requirements of patient confidentiality, using ‘real’ NHS paperwork | 2, 3 |
| 19c | 211) explain their professional and legal responsibilities when accessing information sources in relation to patient care, health promotion, giving advice and information to patients, and research and education | 2, 3 |
| 19d | 212) discuss the role of doctors in contributing to the collection and analysis of patient data at a population level to identify trends in wellbeing, disease and treatment, and to improve healthcare and healthcare system | 2, 3 |
| 19e | 213) apply the principles of health informatics to medical practice | 3 |
| 20a | 214) describe and illustrate from their own professional experience the range of settings in which patients receive care, including in the community, in patients’ homes and in primary and secondary care provider settings | 2, 3 |
| 20b | 215) explain and illustrate from their own professional experience the importance of integrating patients’ care across different settings to ensure person-centred care | 2, 3 |
| 20c | 216) describe emerging trends in settings where care is provided | 2, 3 |
|  | 217) can describe how trauma management is organised in the UK | 2, 3 |
| 20d | 219) describe the relationship between healthcare and social care and how they interact | 1, 2, 3 |
| 24a | 277) recognise how society influences and determines the behaviour of individuals and groups and apply this to the care of patients | 1, 2, 3 |
| 24b | 278) review the sociological concepts of health, illness and disease and apply these to the care of patients | 1, 2, 3 |
|  | 279) demonstrate understanding of principles and criteria for just and sustainable access to, and provision of, finite healthcare resources | 2, 3 |
|  | 280) describe how far human behaviour is responsible for pathology & how culture affects what is perceived as illness and how it presents | 1, 2 |
| 24c | 281) apply theoretical frameworks of sociology to explain the varied responses of individuals, groups and societies to disease | 1, 2, 3 |
| 24d | 282) recognise sociological factors that contribute to illness, the course of the disease and the success of treatment and apply these to the care of patients − including issues relating to health inequalities and the social determinants of health, the links between occupation and health, and the effects of poverty and affluence | 1, 2, 3 |
|  | 283) demonstrate understanding of and ability to use of Social Prescribing as a tool for health promotion and disease prevention | 2 |
| 24e | 284) explain the sociological aspects of behavioural change and treatment concordance and compliance, and apply these models to the care of patients as part of person-centred decision making | 2, 3 |
|  | 287) can describe the importance of lifestyle on mental health and its impact on treatments including sleep hygiene, nutrition, social interaction, fitness, activity, education occupation, and family and community involvement | 2, 3 |
| 25c | 289) evaluate the environmental, social, behavioural and cultural factors which influence health and disease in different populations | 2, 3 |
| 25d | 290) assess, by taking a history, the environmental, social, psychological, behavioural and cultural factors influencing a patient’s presentation, and identify options to address these, including advocacy for those who are disempowered | 2, 3 |
| 25e | 291) apply epidemiological data to manage healthcare for the individual and the community and evaluate the clinical and cost effectiveness of interventions | 2, 3 |
|  | 292) can describe the public health importance of mental health nationally and internationally in terms of personal, economic and social functioning, including a knowledge of prevalence, disability, chronicity, carer burden, cultural attitudes and differences, suicide, and service provision | 3 |
| 25f | 293) outline the principles underlying the development of health, health service policy, and clinical guidelines, including principles of health economics, equity, and sustainable healthcare | 1, 3 |
|  | 296) recognises why preventive medicine provides some of the most strongly evidence-based interventions in medicine and how general-practice records facilitates and enhances the provision of personal preventive medicine. Can describe the value of common preventative measures e.g. folic acid, immunisation | 1, 3 |
| 25k | 304) evaluate the determinants of health and disease and variations in healthcare delivery and medical practice from a global perspective and explain the impact that global changes may have on local health and wellbeing | 2, 3 |
