# Supplementary material for: Framework for implementing asylum seekers and refugees’ health into the undergraduate medical curriculum in the United Kingdom
Source: Health Educ Res. 2024 Jan 25;39(2):170–81. doi: 10.1093/her/cyae002 (PMC10952400; doi:10.1093/her/cyae002)
Supplement: cyae002_Supp [file cyae002_supp.zip › Supp/Supplementary 4.docx]

# **Supplementary table 4. Mapping GMC Learning Outcomes to Global Health Learning Outcomes**

| **Global Health Themes** | **Global Health Learning Outcomes (specific to asylum seekers’ and refugees’ health)** | **Outcomes from *Outcomes for graduates*** |
| --- | --- | --- |
| Global burden of disease | 1. Discuss the impact of international travel and migration on the diseases seen in the UK   2.1 Take an appropriate travel history and recognise common causes of illness in a returning traveller.  2.2 Discuss the aetiology, clinical presentation and management of diseases linked to migration, basing judgement on clinical evidence rather than prejudicial assumption.  2.3 Discuss the basis for the use of immunisations for international travellers and migrant communities in the UK. | 5d, 25k |
| Socioeconomic and environmental determinants of health | 1. Demonstrate awareness of the non-clinical determinants of health, including social, political, economic, environmental, and gender disparities | 6a, 7h, 14a, 20d, 23b, 24b, 24d, 25c, 25d |
| Health systems | 1. Recognise that health systems are structured and function differently across the globe   8.3 Discuss the involvement of multinational corporations and foreign health systems in delivering health care to UK patients | 25f |
| Global health governance | 1. Demonstrate awareness of the complexity of global health governance, including the roles of international organisations, the commercial sector, and civil society 2. Discuss the role of WHO as the international representative body of national governments for health   12.1 Describe the functions of WHO concerning international health policy, disease surveillance, data collection, sharing best practice and setting international norms | 5d, 25f, 25g |
| Human rights and ethics | 1. Respect the rights and equal value of all people without discrimination and provide compassionate care for all   14.1 Respect patient values and beliefs relating to their health, treatment and end of life care.   1. Examine how international legal frameworks impact on health care delivery in the UK 2. Discuss and critique the concept of a right to health   16.1 Discuss the definition of a human right.  16.2 Discuss and critique how the concept of a right to health impacts on health care delivery in the UK.   1. Describe the particular health needs of vulnerable groups and migrant   17.1 Describe the key health needs of refugees, asylum seekers and undocumented migrants in the UK from biomedical, psychological and social perspectives, and how these change over time.  17.2 Recognise that vulnerable groups are protected by specific legal frameworks.   1. Discuss the role of doctors as advocates for their patients, including the importance of prioritising health needs over other concerns and adhering to codes of professional conduct | 14l, 10a, 10b  4  7i  7a, 7c  25d |
| Cultural diversity and health | 1. Demonstrate understanding that culture is important and may influence behaviour, while acknowledging the dangers of assuming that those from a particular social group will behave in a certain way, including relating to:   19.1 health-seeking behaviour 19.2 the doctor-patient relationship 19.3 the use of alternative medicines and treatments 19.4 lifestyle and substance misuse   1. Communicate effectively with people from different ethnic, religious, and social backgrounds, where necessary using external help   20.1 Describe how to access external help for translation, including translation services and leaflets in an appropriate language, and recognise how this can impact communication.  20.2 Identify potential ethical concerns relating to the use of family members as translators.  20.3 Conduct a consultation and examine patients, demonstrating sensitivity to different backgrounds.  20.4 Access information about the impact of a specific background on health risks. | 7a, 14c, 14j, 14l,14m, 23b, 24a, 24b, 24c, 24d, 24e, 18m, 25c  7a, 10a, 10b, 14c, 14j, 14l, 18m |
