# Supplementary material for: Framework for implementing asylum seekers and refugees’ health into the undergraduate medical curriculum in the United Kingdom
Source: Health Educ Res. 2024 Jan 25;39(2):170–81. doi: 10.1093/her/cyae002 (PMC10952400; doi:10.1093/her/cyae002)
Supplement: cyae002_Supp [file cyae002_supp.zip › Supp/Supplementary table 1.doc]

# **Supplementary table 1: List of scoping review search terms.**

| **Database: PubMed**  **Date: 28/10/2022** | **Search words** | **Number of records** |
| --- | --- | --- |
| 1 | (asylum seekers[Title]) AND (medical school[Title]) | 1 |
| 2 | (asylum seekers[Title]) AND (medical curriculum[Title]) | 0 |
| 3 | (refugee[Title]) AND (medical school[Title]) | 0 |
| 4 | (refugee[Title]) AND (medical curriculum[Title]) | 0 |
| 5 | refugee health[Title] | 130 |
| 6 | asylum seeker health [Title] | 9 |
| 7 | (refugee[Title]) AND (medical student[Title]) | 6 |
| 8 | (asylum seekers[Title]) AND (medical student[Title]) | 0 |
| 9 | (refugee[Title]) AND (undergraduate[Title]) | 2 |
| 10 | (asylum seekers[Title]) AND (undergraduate[Title]) | 0 |
| 11 | (refugees[Title]) AND (medical[Title]) | 78 |
| 12 | (asylum seekers[Title]) AND (medical[Title]) | 34 |
| 13 | (homeless[Title]) AND (curriculum[Title]) | 5 |

| **Database: BMC**  **Date: 28/10/2022** | **Search words** | **Number of records** |
| --- | --- | --- |
| 1 | ****Refugee undergraduate medical curriculum**** | 56 |
| 2 | ****Asylum seeker undergraduate medical curriculum**** | 14 |
